# Manic episode, delusional disorder or dementia: due to a case

**DOI:** 10.1192/j.eurpsy.2026.11571

**Published:** 2026-07-31

**Authors:** M. Ríos Vaquero, A. Monllor Lazarraga, G. Lorenzo Chapatte, L. Rojas Vázquez, M. P. Pando Fernández, Ó. Martín Santiago, G. Medina Ojeda

**Affiliations:** Psichiatry, Hospital Clínico Universitario de Valladolid, Valladolid, Spain

## Abstract

**Introduction:**

Elderly patients pose a challenge in clinical practice. These patients may present symptoms that can be due to age-related cognitive impairment or may be due to other pathologies such as late psychosis, delusional disorder or manic episode with psychotic symptoms. It is for this reason that it is important to perform complementary tests and make a good differential diagnosis, with the aim of early treatment and improve evolution and prognosis.

**Objectives:**

The objective is to perform a literature review based on a case of an elderly patient.

**Methods:**

To perform a literature review based on a case of an elderly patient.

**Results:**

Case: 70-year-old woman who lives with her husband and has two daughters. Currently retired. The patient has been in psychiatric consultations for approximately 30 years with a diagnosis of recurrent depressive disorder and under sertraline treatment. She goes to the emergency room with her daughter, as they have noticed a change in her behaviour over the last few days. The daughter reports that she is expansive, irritable, aggressive, who says that the neighbors are envious and watch him, performs many activities, etc. She comments that before her mother was the opposite, whiny and unwilling to do anything. The patient reports that her neighbors are envious of her and want to annoy her with great emotional repercussion and that she is aggressive with her husband because they do not get along. Hospital admission for clinical stabilization.

Examination and complementary tests: A neuropsychological evaluation of cognitive impairment was carried out, which resulted in the onset of cognitive impairment. Test of the watch with alterations and MOCA 25/30.

Evolution: During hospitalization, the patient is initially more expansive, irritable towards the family and with ideas of prejudice and self-referential to neighbors. She also has memory failures. Treatment with oral risperidone is adjusted with good tolerance, after which delirium symptoms are remitting.

**Conclusions:**

The onset of cognitive decline may be accompanied or preceded by delusional symptomatology. It is recommended to perform differential diagnosis with mania, delirious disorder or onset of cognitive decline. Treatment with risperidone or quetiapine is useful.

**Disclosure of Interest:**

None Declared

